# Odorant receptor structures predict the major female sex pheromone component in a moth

**DOI:** 10.1186/s12915-026-02717-1

**Published:** 2026-09-21

**Authors:** Arthur Comte, Zhiqiang Wei, Hui Li, Wen-Zhe Xing, Riccardo Moracci, Jin Zhang, Sébastien Fiorucci, Emmanuelle Jacquin-Joly

**Affiliations:** 1 Université Côte d’Azur, CNRS, ICN, Nice, France; 2 INRAE, Sorbonne Université, CNRS, IRD, Université Paris Cité, Université Paris Est Créteil Val de Marne, Institut d’Ecologie Et Des Sciences de L’Environnement de Paris (iEES-Paris), Versailles, France; 3 Department of Entomology, College of Plant Protection, Nanjing Agricultural University, Nanjing, China

**Keywords:** *Spodoptera picta*, Sex pheromone, Pheromone receptors, In silico functional prediction, Behavioral assays

## Abstract

**Background:**

Species isolation in insects can be ensured by prezygotic barriers, such as species-specific sex pheromone blends that mediate sexual attraction and mate recognition. Yet, sex pheromone compositions of many species remain poorly characterized outside important agricultural pests, because of the necessity to access live insects for pheromone extraction, the difficulties in identifying the production period, and the precise identification of behaviorally relevant compounds in extracts.

**Results:**

Here, we use an odorant receptor (OR)-guided approach to identify the major female sex pheromone component of the previously unexplored lily moth, *Spodoptera picta*. Genome analysis identified *S. picta* orthologs of the pheromone receptors OR5 and OR75, previously characterized in *S. litura* and *S. littoralis*. Structural modeling of OR5 and OR75 combined with molecular docking predicted conserved ligand binding to (*Z*,*E*)-9,11–14:OAc, which was confirmed by functional assays on the ORs. Gas chromatography–mass spectrometry analyses of female pheromone gland extracts identified this compound as the major pheromone component, and electrophysiological and behavioral assays further demonstrated that it elicits antennal responses and is sufficient to attract males.

**Conclusions:**

Together, these results establish an innovative receptor structure-based strategy for pheromone identification in non-model species, provide insights into the sexual chemical ecology of *S. picta*, and clarify the evolutionary pathway by which a unique class of pheromone receptors emerged by gene duplication and gained specificity.

**Supplementary Information:**

The online version contains supplementary material available at https://doi.org/10.1186/s12915-026-02717-1.

## Background

For reproductive success, insects must efficiently locate appropriate mates. To maximize this success, many species, such as moths (Lepidoptera), have evolved a highly specialized chemosensory communication system based on sexual signals, the sex pheromones. In most moth species, the sex pheromone blend is synthesized and released by adult females within a species-specific time window, usually during the scotophase. Adult males detect this blend over long distances and at remarkably low concentrations, thereby initiating attraction behaviors [1]. The composition of the sex pheromone blend, in terms of both constituent molecules and their precise ratios, determines species specificity and contributes to reproductive isolation among sympatric species [2]. Nevertheless, in some cases, the major component of the female sex pheromone blend alone can elicit male attraction [3–5].

Yet, the sex pheromones of many major agricultural pest moth species have been identified [6], primarily because of their potential in pest control strategies, such as population monitoring via sex pheromone trap, mass trapping, or mating disruption through diffusion of synthetic pheromone in the air [7]. In contrast, non-pest or minor pest species have been largely overlooked. This is the case for the lily moth *Spodoptera picta*, whose larvae are specialized feeders on certain plants of the Amaryllidaceae family [8]. Damage caused by *S. picta* larvae is largely restricted to ornamental plants in public and private gardens, where synthetic pesticide use is limited. Identification of components of this species sex pheromone blend could facilitate the development of targeted pheromone-based control strategies.

The sex pheromone blends of two related species of *S. picta*, namely *S. litura* and *S. littoralis*, are well characterized [9]. Both species share the same major component, (*Z*,*E*)−9,11-tetradecadienyl acetate (hereafter abbreviated as (*Z*,*E*)−9,11–14:OAc), which is both necessary and sufficient to elicit adult male attraction [3, 4]. Notably, this acetate seems to be absent from the pheromone blends of all other studied *Spodoptera* species [6]. At the molecular level, the detection of (*Z*,*E*)−9,11–14:OAc is mediated in both *S. litura* and *S. littoralis* by two pheromone receptors, OR5 and its paralog OR75 [10, 11]. Pheromone receptors constitute a specialized subfamily of insect odorant receptors (ORs) dedicated to the recognition of sex pheromone components. Like other ORs, they are seven-transmembrane domain proteins [12–14] expressed in the membranes of olfactory sensory neurons (OSNs) located within specialized olfactory organs, typically the antennae. In neopteran insects, ORs function as heterotetrameric ion channels in association with an obligatory co-receptor, Orco [15–17]. Within these complexes, the highly divergent OR subunit determines ligand specificity and sensitivity by directly binding molecules, whereas the highly conserved Orco subunit is essential for receptor trafficking, complex assembly, and maintenance of the structural integrity of the ion channel.

Although both OR5 and OR75 orthologs in *S. litura* and *S. littoralis* detect (*Z*,*E*)−9,11–14:OAc, the attraction of adult males to this major female sex pheromone component appears to be primarily mediated by OR5. OR5 orthologs in both species are narrowly tuned to (*Z*,*E*)−9,11–14:OAc and exhibit high expression levels in adult male antennae, whereas OR75 orthologs function as broadly tuned receptors with low expression in adult male antennae [11]. Consistently, OR5 knock-out mutants of *S. littoralis* adult males lose their attraction to this compound [10]. We therefore hypothesized that the OR5 ortholog in *S. picta*, if it exists, would also be the receptor to the main pheromone component of *S. picta* female sex pheromone blend. Functional characterization of this putative PR could thus provide the first insight into the composition of the *S. picta* female sex pheromone.

Furthermore, OR75 orthologs—that consist of a duplication of OR5—are found only in *S. litura* and *S. littoralis* and not in other related *Spodoptera* species such as *S. frugiperda* and *S. exigua* [11]. Functional characterization of the putative OR5 and OR75 orthologs in *S. picta* could thus provide critical insight into the unique evolutionary trajectory of the OR5/75 PR clade across the genus *Spodoptera*.

Taking advantage of the recently assembled chromosome-level genome of *S. picta* [18], we confirmed the existence of an OR5 ortholog and its paralog OR75 in *S. picta*. Quantitative PCR analysis revealed over-expression of OR5 compared to OR75 in *S. picta* adult male antennae. Structural modeling of these newly identified receptors, followed by physicochemical characterization of their predicted binding region and molecular docking, suggested that both function as pheromone receptors with ligand selectivity comparable to their orthologs in *S. litura* and *S. littoralis*. Consistent with the in silico predictions, electrophysiological recordings demonstrated that *S. picta* OR5 was narrowly tuned to (*Z*,*E*)−9,11–14:OAc, whereas *S. picta* OR75 exhibited broader selectivity to multiple components of *Spodoptera* sex pheromone blends, including (*Z*,*E*)−9,11–14:OAc. Further gas chromatography (GC) coupled to mass spectrometry (GC–MS) analysis of *S. picta* female sex pheromone gland extracts confirmed that (*Z*,*E*)−9,11–14:OAc—the ligand of *S. picta* OR5—was indeed the major component. Moreover, GC coupled to electroantennographic detection (GC-EAD), electroantennography (EAG), and behavioral assays demonstrated that this compound elicited a strong antennal response and was sufficient to trigger male attraction, confirming we have identified the main component of *S. picta* female sex pheromone blend.

## Results

### OR5 and OR75 orthologs are expressed in adult male antennae of *S. picta*

Using BLAST searches on the published *S. picta* genome [18] with pheromone receptor sequences from *S. exigua*, *S. frugiperda*, *S. litura*, and *S. littoralis* as queries, we successfully annotated the genes encoding orthologs of OR5 and OR75 in *S. picta*, further referred as SpicOR5 and SpicOR75. The predicted SpicOR5 and SpicOR75 proteins comprised 397 amino acids, a length conserved with their orthologs across other *Spodoptera* species. The presence of both start and stop codons in the gene models confirmed that the annotated sequences were complete (Additional file 1: Fig. S1a).

**Fig. 1:**
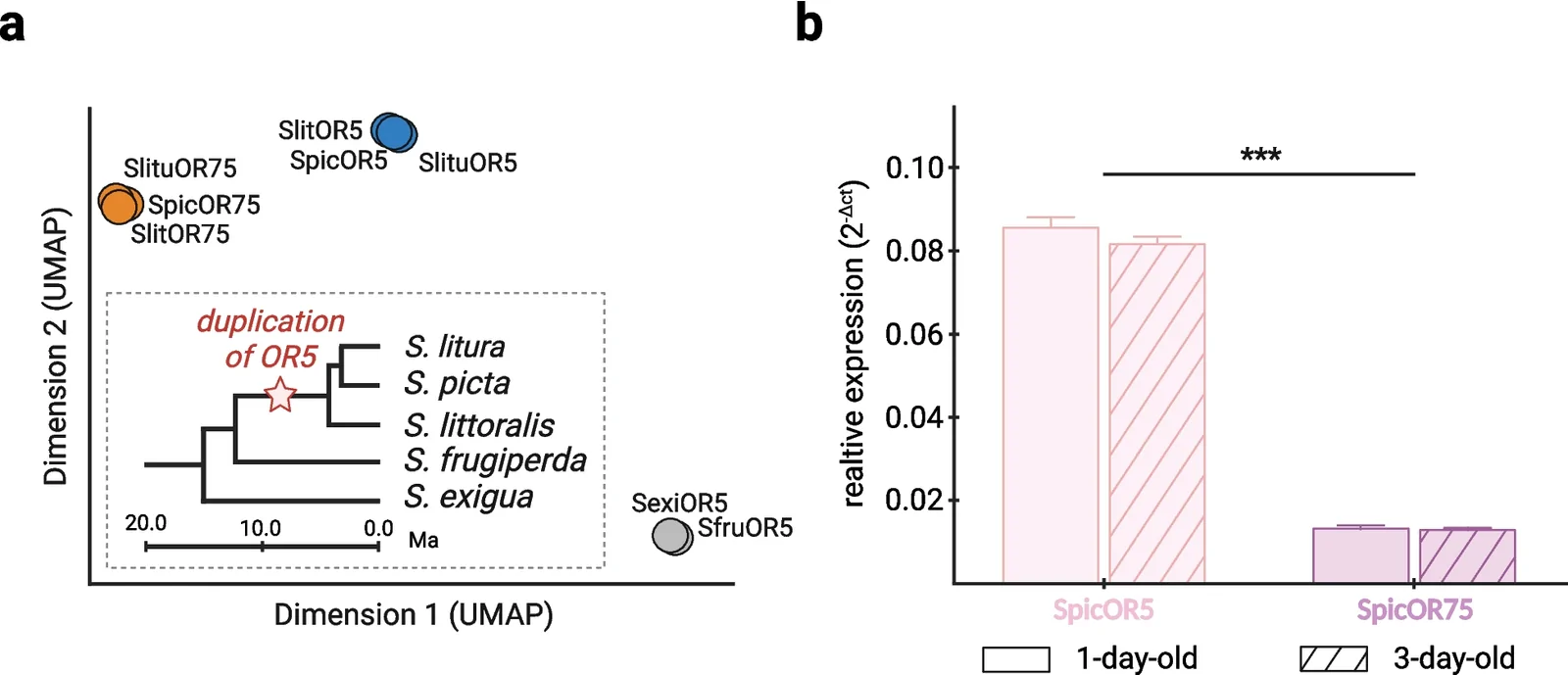
OR75 and OR5 are present in the *S. picta* genome. Among the two copies, SpicOR5 is expressed at a higher level than its paralog, SpicOR75, in adult male antennae. **a** Clustering of OR5 orthologs and paralogs from *S. picta* (Spic), *S. litura* (Slitu), *S. littoralis* (Slit), *S. frugiperda* (Sfru), and *S. exigua* (Sexi) based on their amino acid sequences. Each sequence was one-hot encoded into a numerical vector and dimensionally reduced using UMAP [19] to visualize sequence relationships. Receptors are colored by cluster assignment from HDBSCAN [20], with unclustered receptors shown in gray. A phylogenetic tree of the studied species is shown in the bottom left corner of the plot. Evolutionary relationships and divergence times were extracted from Kergoat et al. [21]. The duplication event leading to OR5 and OR75 is marked with a red star. **b** Relative expression levels of SpicOR5 and SpicOR75 in *S. picta* adult male antennae, measured by RT-qPCR. Two ages were tested: 1-day-old (empty bars) and 3-day-old males (hatched bars). Data are presented as mean relative expression ± SEM (*n* = 3). Asterisks indicate statistically significant differences between the expression levels (***, *p* < 0.001). Expression levels were analyzed using a two-way ANOVA with receptor identity and moth age as factors. A significant effect of receptor identity was detected, whereas neither moth age nor the receptor identity × moth age interaction was significant

SpicOR5 and SpicOR75 shared a high percentage of identity with their orthologs in *S. littoralis* and *S. litura*, with average sequence identities between orthologs of 95.3% and 94.9%, respectively. In contrast, they showed substantially lower identity to *S. exigua* OR5 (67.0% for SpicOR5 and 69.8% for SpicOR75) and *S. frugiperda* OR5 (71.3% and 71.8%, respectively), highlighting their marked divergence from these ancestral sequences (Fig. 1a).

We next assessed the expression levels of the newly identified SpicOR5 and SpicOR75 in adult male antennae using quantitative real-time PCR (RT-qPCR). Consistent with observations in *S. littoralis* and *S. litura* [11], SpicOR5 was highly expressed relative to its paralog SpicOR75 in adult male antennae (Fig. 1b). The expression levels of both genes remained stable between 1-day-old and 3-day-old male moths, suggesting that their expression is not regulated by sexual maturation during early adult life (Fig. 1b).

### Conserved physicochemical properties of binding regions suggest preserved function of OR5 and OR75 orthologs in *S. picta*, *S. litura*, and *S. littoralis*

**Fig. 2:**
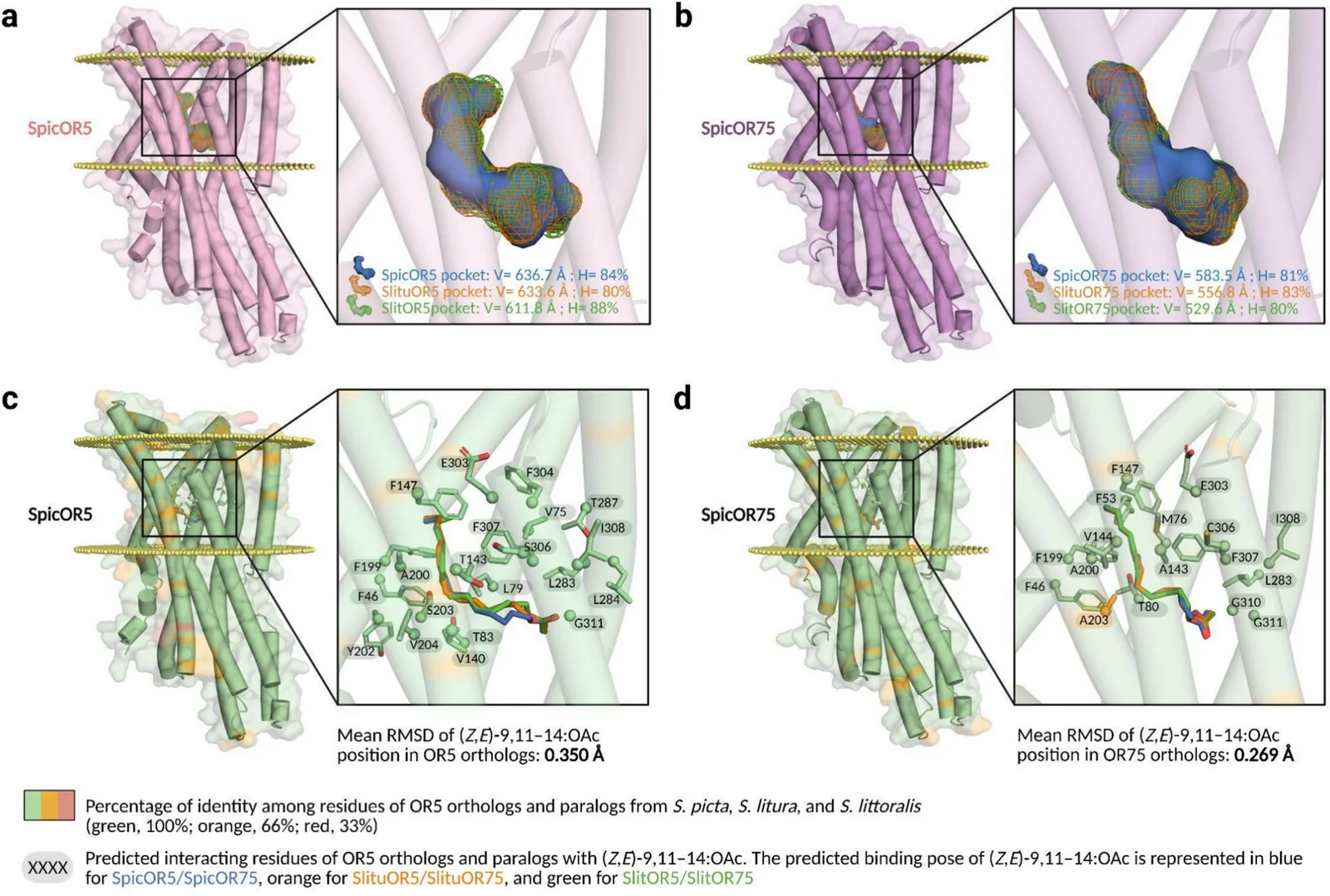
The highly conserved binding regions of OR5 orthologs and paralogs from *S. picta*, *S. litura*, and *S. littoralis* suggest conserved ligand tuning across the three sister species. **a** SpicOR5 and **b** SpicOR75 AlphaFold 3 models [22], each shown with predicted binding pockets corresponding to the receptor itself (blue), and its orthologs in *S. litura* (orange) and *S. littoralis* (green). Cavities were identified and analyzed using Fpocket [23]. “V” indicates the pocket volume and “H” the proportion of apolar spheres in the cavity. 3D models of SpicOR5 (**c**) and SpicOR75 (**d**), each showing the top-ranked docking pose of (*Z*,*E*)−9,11–14:OAc in SpicORs (blue), *S. litura* orthologs (orange), and *S. littoralis* orthologs (green). Side chains of all residues predicted to interact with the ligand—identified across SpicOR, SlituOR, and SlitOR from the nine best-scoring poses—are displayed. Residue-by-residue sequence identity among orthologs was mapped using a color gradient, with fully conserved positions (100%) shown in green, partially conserved positions (66%) in orange, and non-conserved positions (33%) in red. Receptor orientation in the membrane was determined using the PPM 3.0 web server [24]. This figure was generated using the molecular visualization software PyMol [25]

We next generated three-dimensional models of OR5 orthologs and paralogs from *S. picta*, *S. litura*, *S. littoralis*, *S. frugiperda*, and *S. exigua* using the AlphaFold 3 web server [22]. The high sequence conservation between SpicOR5, SpicOR75, and their orthologs in *S. litura* and *S. littoralis* was mirrored by strong structural similarity, with mean RMSD values of 0.308 Å for OR5 and 0.288 Å for OR75, calculated across the full-length modeled proteins. This global structural conservation extended locally to the predicted binding regions. Fpocket analysis revealed comparable cavity volumes and cavity hydrophobicity among all orthologs (Fig. 2a, b). The conservation of these physicochemical properties suggests that ligands may interact with these receptors through similar binding modes.

Molecular docking of (*Z*,*E*)−9,11–14:OAc—the only known ligand for SlitOR5 and SlituOR5 [11]—predicted a conserved binding pose within the binding regions of SpicOR5, SlituOR5, and SlitOR5. (*Z*,*E*)−9,11–14:OAc consistently engaged conserved amino acid residues shared among the three sister species (Fig. 2c), and the mean RMSD between docked ligand positions was 0.350 Å. These results led us to hypothesize that SpicOR5 is tuned to recognize a chemical space comparable to that of its orthologs in *S. litura* and *S. littoralis*, with specific affinity for (*Z*,*E*)−9,11–14:OAc.

Similarly, molecular docking of the eight known ligands for SlituOR75 and SlitOR75—(*Z*,*E*)−9,11–14:OAc, (*Z*,*Z*)−9,11–14:OAc, (*E*)−11–14:OAc, (*E*,*E*)−9,12–14:OAc, 14:OAc, (*Z*,*E*)−9,12–14:OAc, 12:OAc, and (*E*)−7–12:OAc [11]—revealed comparable binding poses across all three OR75 orthologs. Only one residue predicted to interact with at least one of the eight pheromone components differed among species: at position 203, SpicOR75 and SlituOR75 carried a serine, whereas SlitOR75 carried an alanine (Fig. 2d, Additional file 1: Fig. S1a). This substitution appeared to have minimal impact on ligand binding specificity, as both SlituOR75 and SlitOR75 are known to function as broadly tuned receptors and share largely overlapping ligand repertoires [11]. We thus hypothesized that SpicOR75 functions as a broadly tuned receptor, capable of detecting a diverse range of pheromone components similarly to its orthologs in *S. litura* and *S. littoralis*.

### Single-sensillum recordings confirm the predicted tuning of SpicOR5 and SpicOR75

To validate ligand predictions based on the analysis of putative binding regions of SpicOR5 and SpicOR75, we generated transgenic *Drosophila melanogaster* expressing these receptors in at1 OSNs, replacing the endogenous DmelOR67d [26]. Single-sensillum recordings were then conducted upon stimulation with a panel of 26 moth female sex pheromone compounds that has been previously used to functionally characterize *S. litura* and *S. littoralis* OR5 and OR75 [11].

**Fig. 3:**
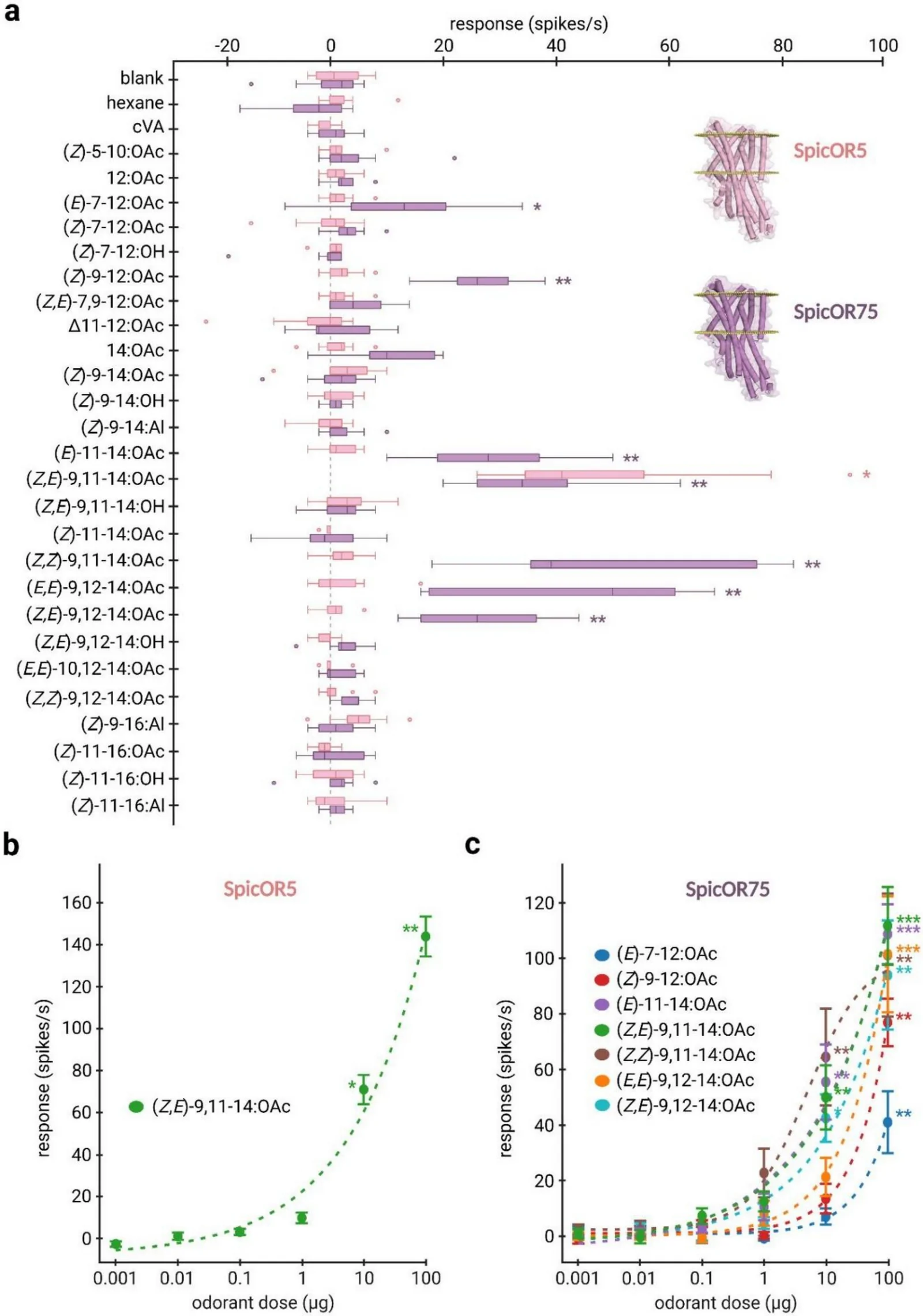
The pheromone receptors OR5 and OR75 exhibit a conserved function in *S. picta*, *S. litura*, and *S. littoralis*. SpicOR5 is narrowly tuned and specifically responsive to (*Z*,*E*)−9,11–14:OAc, while SpicOR75 functions as a broadly tuned pheromone receptor. **a** Box plots show the responses of *Drosophila* at1 OSNs expressing SpicOR5 (pink) or SpicOR75 (purple) to controls (blank, hexane, cVA) and a panel of 26 moth pheromone compounds (10 µg per stimulus, *n* = 8). The whiskers were extended to 1.5 times the interquartile range from the quartiles. Outliers are indicated by dots. Dose–response curves of *Drosophila* at1 OSNs expressing SpicOR5 (**b**) and SpicOR75 (**c**) in response to ligands identified from the initial screening in **a**. Data are shown as mean spike frequencies ± SEM (*n* = 8). Dose–response data were fitted with sigmoidal curves when a plateau was reached, or with exponential functions otherwise, using the curve_fit function from the SciPy 1.16.0 library. In **a**–**c**, asterisks indicate statistically significant differences between responses to the odorant and to the solvent (Friedman test followed by Dunn’s post hoc test with Benjamini and Yekutieli correction for multiple comparisons; *adjusted *p* < 0.05, **adjusted *p* < 0.01, ***adjusted *p* < 0.001)

Consistent with the function of its orthologs in *S. litura* and *S. littoralis*, SpicOR5 appeared to be specifically tuned to (*Z*,*E*)−9,11–14:OAc. Among the tested panel, (*Z*,*E*)−9,11–14:OAc was the only compound that significantly activated SpicOR5, eliciting a median response of 49 ± 8.3 spikes/s in neurons expressing this receptor (Fig. 3a). In dose–response experiments, SpicOR5 was significantly activated by (*Z*,*E*)−9,11–14:OAc starting at 10 µg (Fig. 3b). This result supports our prediction based on the comparative analysis of the putative binding regions of OR5 orthologs.

As predicted, SpicOR75 functioned as a broadly tuned receptor, capable of detecting a diverse range of pheromone compounds: seven of the 26 tested molecules elicited significant responses (Fig. 3a). SpicOR75 responded to a ligand set shared with its orthologs SlituOR75 and SlitOR75, including (*E*)−7–12:OAc, (*E*)−11–14:OAc, (*Z*,*E*)−9,11–14:OAc, (*Z*,*Z*)−9,11–14:OAc, (*E*,*E*)−9,12–14:OAc, and (*Z*,*E*)−9,12–14:OAc. The highest responses were evoked by (*E*,*E*)−9,12–14:OAc and (*Z*,*Z*)−9,11–14:OAc, which induced median firing rates of 43 ± 8.0 and 50 ± 8.7 spikes/s, respectively. Dose–response experiments were performed for the seven pheromone compounds that elicited significant activation of at1 OSNs expressing SpicOR75 during the initial screening. SpicOR75 exhibited higher sensitivity to (*E*)−11–14:OAc, (*Z*,*E*)−9,11–14:OAc, (*Z*,*Z*)−9,11–14:OAc, and (*Z*,*E*)−9,12–14:OAc, with neuronal responses starting at 10 µg in the stimulus cartridge (Fig. 3c). In contrast, (*E*)−7–12:OAc, (*Z*)−9–12:OAc, and (*E*,*E*)−9,12–14:OAc required a higher dose of 100 µg to elicit significant activation (Fig. 3c).

### As suspected from SpicOR5 structural and functional analysis, (*Z*,*E*)−9,11–14:OAc is the major component of the *S. picta* female sex pheromone blend

In the previous sections, we demonstrated that SpicOR5, like SlitOR5 and SlituOR5, was specifically tuned to (*Z*,*E*)−9,11–14:OAc. As (*Z*,*E*)−9,11–14:OAc represents the major female sex pheromone component in *S. litura* and *S. littoralis*, and given the conserved function and expression patterns of OR5 orthologs across these three sister species ([11] and this study), we hypothesized that (*Z*,*E*)−9,11–14:OAc similarly constitutes the major female sex pheromone component in *S. picta*. To test this hypothesis, we analyzed adult female pheromone gland extracts, evaluated adult male antennal responses, and assessed adult male sexual behavior.

The optimal time for extracting the female sex pheromone was determined by monitoring, at different ages and throughout the scotophase, the proportion of adult females exhibiting calling behavior, as increased calling activity in moths usually correlates with heightened pheromone emission [27, 28]. *S. picta* adult females displayed peak calling behavior during the first 2 days after adult emergence (Additional file 1: Fig. S2a). After this period, the frequency of calling declined sharply and was nearly absent at day 5 post-emergence. Moreover, calling behavior was not uniformly distributed across the scotophase. The proportion of calling females remained low during the initial hours of the night, began to increase approximately 5 h after the onset of darkness, and peaked between 6.5 and 9 h into the scotophase, with up to 60% of both 1- and 2-day-old females exhibiting calling behavior during this period (Additional file 1: Fig. S2a). For subsequent GC-EAD and GC-MS analyses, pheromone glands were thus dissected from 2-day-old adult females between 6 and 9 h after the onset of the scotophase, corresponding to the peak period of calling activity.

**Fig. 4:**
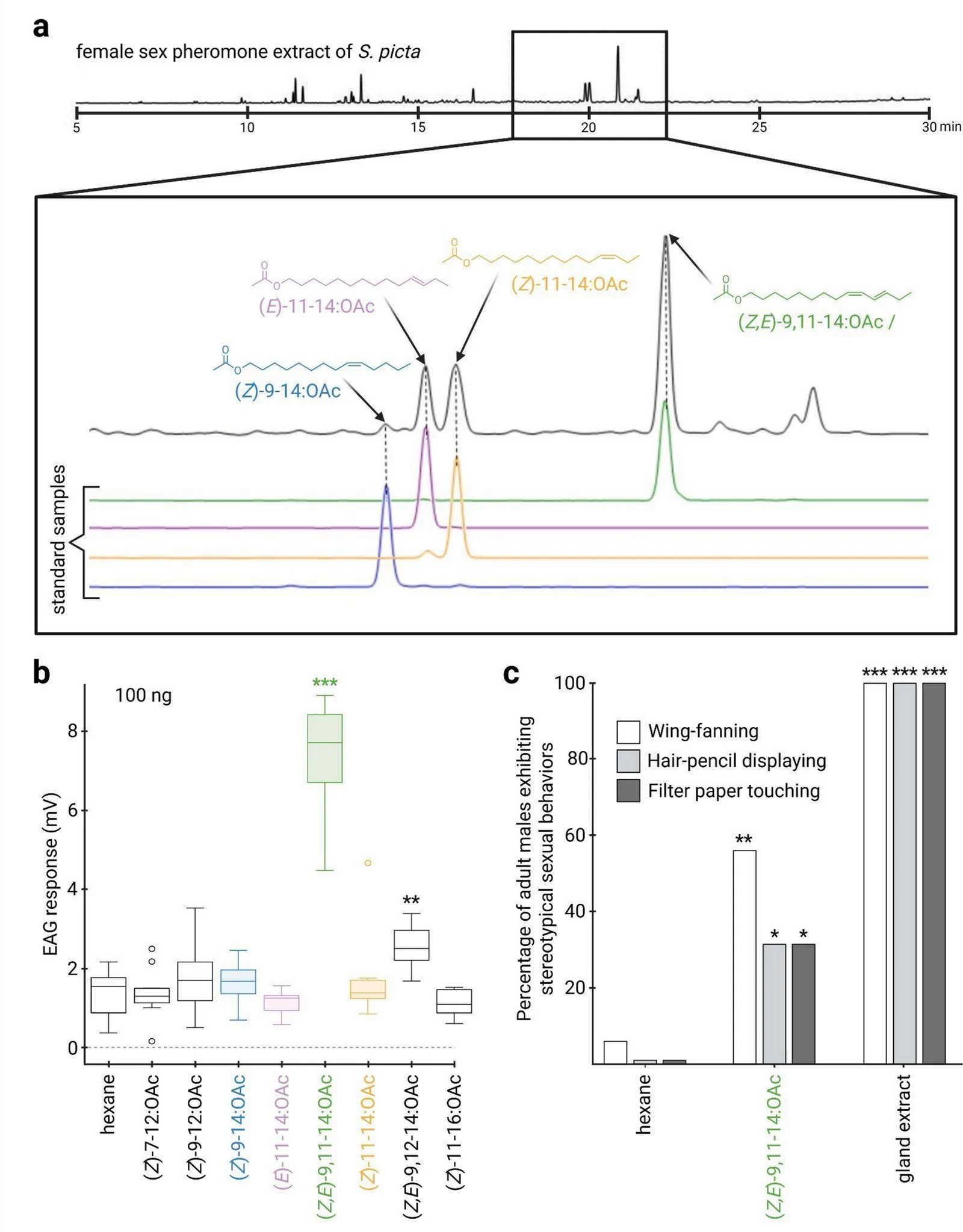
(*Z*,*E*)−9,11–14:OAc is the major component of the *S. picta* female sex pheromone blend. **a** Identification of compounds extracted from sex pheromone glands of *S. picta* adult females. The GC trace of a representative pheromone extract (20 pheromone glands) is aligned with that of synthetic standards (colored traces) that matched peaks in the pheromone extract trace. Names of the identified components are connected to their corresponding peaks with arrows. **b** Box plot shows EAG responses of male *S. picta* antennae to 100 ng of the synthetic representatives of pheromone components identified in **a** and structurally related analogs (*n* = 10). Boxes corresponding to female sex pheromone components are colored as in **a**, whereas boxes for other components are shown in black. Whiskers extend to 1.5 times the interquartile range from the quartiles, with outliers indicated by dots. Asterisks denote statistically significant differences between responses to the odorant and the solvent (hexane) (Friedman test followed by Dunn’s post hoc test with Benjamini and Yekutieli correction for multiple comparisons; **adjusted *p* < 0.01, ***adjusted *p* < 0.001). **c** Bar chart summarizing the male behavioral response to (*Z*,*E*)−9,11–14:OAc (10 µg on filter paper), categorized into three stereotypical sexual behaviors: wing-fanning (white bars), hair-pencil displaying (light gray bars), and filter paper touching (gray bars) [29–31]. Values represent the percentage of responding males (*n* = 16). Hexane was used as a negative control (10 µg on filter paper) and female gland extract as a positive control (one gland excised and applied to filter paper). Asterisks illustrate statistically significant differences between (*Z*,*E*)−9,11–14:OAc or female gland extract and the negative control (generalized linear mixed model: response ~ compound tested × stereotypical sexual behaviors + (1|Male_ID), followed by multiple-comparison post hoc tests with a Bonferroni correction; *adjusted *p* < 0.05, **adjusted *p* < 0.01, ***adjusted *p* < 0.001)

Following collection, the female sex pheromone extracts were analyzed using GC-EAD. Among all components separated by GC, only one elicited antennal response in 10 out of the 18 tested male antennae (Additional file 1: Fig. S2b). This compound corresponded to the most prominent peak in the GC trace and was therefore considered as the major component of the female sex pheromone extract. Subsequent GC-MS analysis, performed alongside synthetic standards, identified this compound as (*Z*,*E*)−9,11–14:OAc (Fig. 4a). Additionally, three minor components were detected in the female pheromone blend: (*Z*)−9–14:OAc, (*E*)−11–14:OAc, and (*Z*)−11–14:OAc (Fig. 4a). The relative ratio of these components in the female gland extract was 100:45:34:3 for (*Z*,*E*)−9,11–14:OAc, (*Z*)−9–14:OAc, (*E*)−11–14:OAc, and (*Z*)−11–14:OAc, respectively.

To confirm antennal detection of the identified compounds, synthetic standards were individually tested using EAG on adult *S. picta* males. Structurally related analogs were included in the test panel. At a dose of 100 ng, only (*Z*,*E*)−9,11–14:OAc and its analog (*Z*,*E*)−9,12–14:OAc elicited significant antennal responses, with mean amplitudes of 7.109 mV and 2.542 mV, respectively (Fig. 4b). (*Z*,*E*)−9,11–14:OAc induced the strongest EAG response, supporting its role as the major sex pheromone component. When the dose was increased to 1000 ng, (*Z*)−9–14:OAc also elicited a significant antennal response (Additional file 1: Fig. S3).

Finally, we assessed adult male behavioral responses to (*Z*,*E*)−9,11–14:OAc. Three stereotypical sexual behaviors—previously reported in the genus *Spodoptera*—were recorded, each representing a distinct step in pheromone recognition: wing-fanning, hair-pencil display, and odor-source contact [29–31]. (*Z*,*E*)−9,11–14:OAc alone was sufficient to elicit adult male attraction. At 10 µg on the filter paper, 56.3% of the 16 males exhibited wing-fanning, while 31.3% performed hair-pencil display and touched the odor source (Fig. 4c). We then tested a synthetic blend containing the major and minor components identified by GC-MS analysis of the female gland extract in a similar ratio. Compared to (*Z*,*E*)−9,11–14:OAc alone, the addition of the minor components elicited more stereotypical male sexual behaviors: at 10 µg on filter paper, 64.7% of the 17 males exhibited wing-fanning, while 58.8% performed hair-pencil display and contacted the odor source (Additional file 1: Fig. S4). Nevertheless, the synthetic blend still elicited less stereotypical male sexual behaviors than a sex pheromone gland extract, for which 100% of the males presented all behavioral items (Fig. 4c).

## Discussion

In this study, we not only provided the first insights into the sexual chemical ecology of the non-model species *S. picta* by identifying its major female sex pheromone component, but also demonstrated that an in silico OR-guided approach can successfully help to identify unknown moth sex pheromones. Whereas all moth sex pheromone identifications so far relied on sex pheromone gland extraction or headspace collection from alive insects and further analytical and behavioral analyses, necessitating access to alive insects, we here used a novel reverse chemical ecology approach in which in silico predictions guided electrophysiological and behavioral assays.

In the recently published genome of *S. picta* [18], we identified orthologs of OR5 and OR75, two ORs tuned to the main female sex pheromone component in the *S. picta* sister species, *S. litura* and *S. littoralis* [11]. The strong expression of SpicOR5 in male antennae mirrored that of its orthologs, suggesting a key role in sexual communication and potentially in the detection of the yet unidentified major female sex pheromone component of *S. picta*. Indeed, in moth species, pheromone receptors most highly expressed in adult male antennae often respond to the major female sex pheromone component, as demonstrated in species such as *S. littoralis* [10], *S. frugiperda* [32], and *Helicoverpa armigera* [33].

Altogether, the high sequence identity of OR5 and OR75 orthologs across *S. picta*, *S. litura*, and *S. littoralis*, and the OR5 strong expression in adult male antennae, suggested a conserved function for these pheromone receptors among the three species. However, one or two single amino acid substitutions have been shown to markedly alter pheromone receptor tuning in moths [34–36]. Therefore, to reliably predict the chemical space recognized by the two newly identified *S. picta* OR5 and OR75, we modeled their structures alongside their deorphanized orthologs in *S. littoralis* and *S. litura*, carefully verifying that no spontaneous substitutions affected the physicochemical properties of their binding regions or altered ligand-binding modes. Similar in silico approaches have previously been applied to investigate the function of OR paralogs in mammals [37] and, more recently, orthologous pheromone receptors in insects [38, 39]. However, our approach provides a more comprehensive analysis. In addition to examining the conservation of residues predicted to constitute the binding region, we evaluated both the volume and hydrophobicity of this critical region—two physicochemical parameters shown to be directly linked to OR tuning [40]. Using molecular docking, we demonstrated that known ligands of SlituOR5/SlitOR5 and SlituOR75/SlituOR75 were predicted to adopt similar binding poses within the binding regions of their corresponding orthologs in *S. picta*. Collectively, these in silico analyses supported our hypothesis of a conserved function among OR5 and OR75 orthologs across *S. picta*, *S. litura*, and *S. littoralis*. We validated these predictions through functional assays, demonstrating the robustness of our in silico analysis.

Based on these data, we proposed that *S. picta* uses (*Z*,*E*)−9,11–14:OAc as its major female sex pheromone component. This hypothesis was further validated combining pheromone gland analyses, electrophysiology, and behavioral experiments conducted on *S. picta*. Thus, starting from the amino acid sequence of pheromone receptors, we successfully identified the major female sex pheromone component of this species. This finding paves the way for the development of pheromone-based targeted pest management strategies to control *S. picta*, whose larvae cause extensive damage to plants in both public and private gardens.

In addition to the major component, we identified three additional constituents of the female pheromone blend of *S. picta*: (*Z*)−9‑14:OAc, (*E*)−11‑14:OAc, and (*Z*)−11‑14:OAc, present in a relative ratio of 100:45:34:3. This blend is unlikely to represent the complete pheromone signal, as the synthetic mixture formulated with these four compounds elicited weaker male attraction than female pheromone gland extracts. This difference suggests that additional behaviorally active compounds, potentially present in trace amounts, contribute to male attraction. A similar situation has been reported in *S. frugiperda*, where the minor component (*Z*,*E*)−9,12–14:OAc, present at only ~ 0.2% of the abundance of the major component in female gland extracts, was shown by electrophysiological and field studies to significantly enhance male responses compared to previously used synthetic blends [41].

Nevertheless, we demonstrated that *S. picta* shares the same major female sex pheromone component (*Z*,*E*)−9,11–14:OAc, with its two sister species *S. litura* and *S. littoralis*. In all three species, this compound alone is sufficient to elicit adult male attraction [3, 4]. In *S. littoralis*, which is geographically restricted to Europe, Africa, and the Middle East [42], the use of this shared pheromone component may not compromise conspecific mate recognition, as *S. picta* and *S. litura* geographical distribution does not overlap with that of *S. littoralis*. In contrast, *S. picta* and *S. litura* are sympatric across large regions of South Asia and Oceania [8, 43], generating a potential risk of interspecific sexual signal interference that could affect reproductive isolation. While sympatric species sharing similar pheromone blends can ensure species isolation through calling at different periods, this does not appear to be the case in *S. picta* and *S. litura*. Indeed, we demonstrated here that *S. picta* calling period corresponded to the second half of the scotophase, as observed in *S. litura* [44]. Thus, the overlap in their main sex pheromone component may have imposed strong selective pressures favoring the evolution of distinct minor female sex pheromone components, modified blend ratios, or alternative mating cues, thereby reinforcing species-specific communication and preserving reproductive isolation. The pheromone blend of *S. litura* is indeed qualitatively and quantitatively different from that of *S. picta*. It is composed of (*Z*,*E*)−9,11–14:OAc, (*E*,*E*)−10,12–14:OAc, (*Z*)−9–14:OAc, and (*Z*,*E*)−9,12–14:OAc at a ratio of 100:23:23:16 [9]. Beyond blend ratio differences, two minor components diverge between the species: adult females of *S. picta* synthesize (*E*)−11–14:OAc and (Z)−11–14:OAc, whereas adult females of *S. litura* emit (*E*,*E*)−10,12–14:OAc and (Z,E)−9,12–14:OAc. These species-specific minor components may serve as critical cues enabling males to discriminate conspecific from heterospecific mates, as has been demonstrated in other *Spodoptera* species. For instance, the addition of (*Z*)−9–14:OH—a pheromone component of *S. exigua*—to the female sex pheromone blends of *S. litura* and *S. littoralis* significantly reduces adult male capture rates in pheromone traps [2, 45]. Similarly, adult males of *S. exigua* exhibit avoidance behavior toward (*Z*,*E*)−9,11–14:OAc [2]. In *S. litura*, SlituOR16 is a sex-biased pheromone receptor highly expressed in adult male antennae that exhibits a modest response to (*E*)−11–14:OAc [46], a minor component we identified in the *S. picta* sex pheromone but not reported in the *S. litura* sex pheromone. Reversely, we showed that *S. picta* adult males responded in EAG to (*Z*,*E*)−9,12–14:OAc, a compound we did not find in *S. picta* sex pheromone but present in *S. litura* sex pheromone [9]. Further behavioral assays are required to determine whether these minor sex pheromone components function as behavioral antagonists in *S. picta* and *S. litura* and contribute to reproductive isolation between these two species. In addition to behavioral antagonists, other cues such as host plant volatiles could also participate in refining sex pheromone communication [47]. For instance, the addition of host plant volatiles to a synthetic pheromone blend more than doubled the attraction of adult male *S. exigua* [48]. Specializing on certain Amaryllidaceae plants, *S. picta* may exploit volatiles present in the headspace of its host plants to enhance sexual behavior, cues that may be not attractive to its polyphagous sister species, *S. litura*.

Regarding the unique evolutionary trajectory of the OR5/75 receptor clade across *Spodoptera* that led to OR5 specialization to (*Z*,*E*)−9,11–14:OAc in *S. litura* and *S. littoralis* [11], the functional characterization of SpicOR5 and SpicOR75 completes the story. In *S. exigua* and *S. frugiperda*, species that do not produce (*Z*,*E*)−9,11–14:OAc in their pheromone blend and that do not possess the OR75 gene in their genome, OR5 orthologs are broadly tuned receptors that mediate recognition of both heterospecific and conspecific female sex pheromone components. Following a gene duplication event, two paralogs—OR5 and OR75—are present in *S. litura* and *S. littoralis*, and we extend this observation in *S. picta*. Multiple amino acid substitutions within the binding regions of OR5 orthologs in these three sister species have shifted receptor specificity toward a narrow recognition of their major female sex pheromone component ([11] and this study). In contrast, OR75 orthologs in these species appear to have largely retained the ancestral function of SfruOR5 and SexiOR5, maintaining broad tuning to both heterospecific and conspecific pheromone components. However, a careful comparison of the response spectra of SpicOR75, SlituOR75, and SlitOR75 with those of SexiOR5 and SfruOR5 revealed notable differences in specificity. For example, several ligands recognized by SexiOR5 and SfruOR5—such as *(Z*)−7–12:OAc, Δ11–12:OAc, (*Z*)−11–14:OAc, (*Z*,*Z*)−9,12–14:OAc, and (*Z*)−11–16:OAc—were not detected by OR75 orthologs in *S. picta*, *S. litura*, and *S. littoralis*. Thus, both OR5 and OR75 orthologs in these species exemplify neofunctionalization relative to the ancestral SexiOR5 and SfruOR5, with OR5 gaining strict specificity and OR75 undergoing only partial neofunctionalization—retaining broad tuning but with altered specificity. Strikingly, this functional divergence is mirrored by structural divergence within their binding regions. Clustering analyses of binding-site properties resolved two well-defined groups: SpicOR5, SlituOR5, and SlitOR5 formed one cluster, whereas SpicOR75, SlituOR75, and SlitOR75 formed another. In contrast, SexiOR5 and SfruOR5 remained outside both clusters (Additional file 1: Fig. S1b).

Interestingly, all *Spodoptera* OR5 and OR75 orthologs described belong to a recently identified new lineage of Lepidoptera sex pheromone receptors [10]. While most of the receptors in this lineage are tuned to minor or heterologous sex pheromone compounds [49, 50], SpicOR5, SlituOR5, and SlitOR5 are critical in mate recognition success as they are specifically tuned to the main component of their respective species sex pheromone blends. Although we shed light on the duplication and neofunctionalization events that led to specific tuning to the main component (*Z*,*E*)−9,11–14:OAc, questions remain about the independent evolution of these critical pheromone receptors from classical main pheromone component receptors in Lepidoptera.

## Conclusions

This study demonstrates that a structure-based, odorant receptor-guided approach successfully identified (*Z*,*E*)−9,11–14:OAc as the major female sex pheromone component of the non-model lily moth *Spodoptera picta*. By integrating AI-driven molecular modeling, transcriptomics, electrophysiological assays, analytical chemistry, and behavioral assays, we established a multidisciplinary workflow that links receptor structure to pheromone function. The predicted pheromone compound was validated through GC-MS analysis of gland extracts and shown to elicit both antennal responses and male attraction of male lily moths, confirming its biological relevance. Together, these results highlight how combining computational, chemical, and neurophysiological methods can accelerate pheromone discovery in previously unexplored species. They also clarify the evolutionary pathway by which the OR5 pheromone receptor clade emerged by gene duplication and gained specificity, while providing an efficient attractant for monitoring and trapping a pest species in areas where chemical pesticides are banned.

## Methods

### *S. picta* rearing

*S. picta* specimens were collected from Hunan, China, and reared on crinum plants (*Crinum asiaticum*, purchased from www.taobao.com) under controlled conditions at 26 ± 1 °C and 60 ± 10% relative humidity, with a 14:10-h light:dark photoperiod. Pupae were sexed and housed separately to obtain virgin adult populations. Upon eclosion, adults were maintained with access to 10% honey solution as a nutritional source.

### Gene annotation and RT-qPCR-based expression analysis of SpicOR5 and SpicOR75

Manually annotated amino acid sequences of OR5 and OR75 orthologs of *S. litura* (Slitu), *S. littoralis* (Slit), *S. frugiperda* (Sfru), and *S. exigua* (Sexi) [11] were used as queries to identify OR5 and OR75 orthologs in the *S. picta* (Spic) genome [18]. Searches were conducted using tblastn 2.14.1 with an *e*-value cutoff of 1 × 10^−4^ [51]. The resulting SpicOR sequences were aligned with their orthologs using the MAFFT 7.220 web server [52]. To evaluate patterns of divergence or convergence among OR5 orthologs and paralogs across the five *Spodoptera* species, each receptor sequence was one-hot encoded, yielding a 397-dimensional vector representing amino acid identities at aligned positions. Dimensionality reduction was performed using UMAP 0.5.4 with parameters n_neighbors = 2 and min_dist = 0.0 [19]. Clustering was subsequently carried out with HDBSCAN 0.8.38, using a minimum cluster size of 2 and default settings [20].

To quantify SpicOR5 and SpicOR75 expression in male *S. picta* antennae, RNA was extracted from three biological replicates for each of two age groups: 1- and 3-day-old adult males. Each replicate comprised 20 pairs of antennae from laboratory-reared insects. Gene-specific primers were designed using Beacon Designer 7 software (PREMIER Biosoft, USA) (sequences provided in Additional file 1: Table S1). RT-qPCR was performed on a QuantStudio™ 6 Flex Real-Time PCR System (Applied Biosystems, USA) in 20 μL reactions containing ChamQ™ Universal SYBR qPCR Master Mix (Vazyme, China), according to the manufacturer’s instructions. Thermal cycling was conducted as follows: initial denaturation at 95 °C for 30 s, followed by 40 cycles of 95 °C for 5 s and 60 °C for 34 s. The reference gene EF1-α (elongation factor 1-alpha) was used for normalization, and relative gene expression levels were calculated using the 2^ − ΔCT method. Expression levels were analyzed using a two-way ANOVA with moth age and receptor identity as factors, performed in R 4.3.2.

### Comparative analysis of the predicted binding region of OR5 and OR75 orthologs across five related *Spodoptera* species

Five relaxed models were generated using AlphaFold 3 web server [22] for the orthologs of OR5 and OR75 from *S. picta*, *S. litura*, *S. littoralis*, *S. frugiperda*, and *S. exigua.* Binding regions were defined by structurally aligning the predicted models with experimentally resolved insect OR cryo-electron microscopy structures [15–17] using PyMOL 2.5.4 [25], followed by cavity detection with Fpocket 4.0 [23]. Residues within 5 Å from the surface of the selected cavity were considered part of the binding region. For each ortholog, the model with the highest overall per-residue confidence score (predicted local distance difference test, pLDDT) was selected. Particular attention was given to the pLDDT values within the predicted binding regions to ensure structural reliability in these functionally critical regions (Additional file 1: Fig. S5). Physicochemical properties of the predicted binding regions, including cavity volume and hydrophobicity, were extracted using Fpocket [23]. Both cavity volume and hydrophobicity have been shown to correlate with OR tuning [40]. Hydrophobicity was quantified as the *apolar α-sphere proportion*, a parameter that is more readily interpretable than alternatives such as mean local hydrophobicity. OR5 orthologs and paralogs from the five studied *Spodoptera* species were clustered based on these two parameters using HDBSCAN [20], with a minimum cluster size of 2 and default settings.

Known ligands of SlituOR5, SlituOR75, SlitOR5, and SlitOR75 [11] were docked into their corresponding OR orthologs in *S. picta*, *S. litura*, and *S. littoralis* using the Vinardo scoring function, implemented in the Smina software (release: October 15, 2019; based on AutoDock Vina 1.1.2). Receptor and ligand preparation followed the standardized protocol described by Comte et al. [40]. The docking grid box was manually defined to encompass all residues identified as part of the predicted binding region (center_x = 10, center_y = − 1.67, center_z = − 12, size_x = 13, size_y = 13.9, size_z = 15). The nine best-scoring docking poses for each ligand were retained for analysis of ligand–receptor interactions using ProLIF 2.0.3 [53]. A residue was considered potentially involved in ligand recognition if it interacted with the ligand in at least one of these top nine poses. Root-mean-square deviation (RMSD) analyses were performed using PyMOL [25] to compare the highest-ranked docking pose of each compound for each receptor.

### Heterologous expression in *Drosophila melanogaster* at1 OSNs

The full-length open reading frames of SpicOR5 and SpicOR75 were synthesized in vitro and subcloned into the pUAST.attB vector (Synbio Technologies, Monmouth Junction, NJ, USA). Coding sequences were codon-optimized for expression in *D. melanogaster* (Dmel). The resulting pUAST.attB-SpicOR constructs were injected into *D. melanogaster* embryos (genotype y^1^ M{vas-int.Dm}ZH-2A w; M{3xP3-RFP.attP}ZH-51C) by BestGene Inc. (Chino Hills, CA, USA) using the PhiC31 integrase system, facilitating site-specific integration into the attP landing site at locus 51 C on the second chromosome. Transgenic UAS-SpicOR5 and UAS-SpicOR75 lines were crossed with the *D. melanogaster* OR67d^GAL4^ line to generate homozygous flies expressing the transgenes in at1 OSNs, replacing the endogenous DmelOR67d [26]. Transgenic flies were reared on standard cornmeal-yeast-agar medium at 25 °C under a 12-h light/dark cycle in a Sanyo MIR-553 incubator.

### Functional characterization of SpicOR5 and SpicOR75

Twenty-four hours prior to single-sensillum recording, transgenic flies were transferred to a Sanyo MIR-553 incubator maintained at 29 °C to enhance GAL4-driven expression while minimizing effects on fly viability. Male and female flies aged 3–5 days were randomly selected. The single-sensillum recording procedures and equipment were implemented following the methodology described in Comte et al. [40].

The screening panel of moth pheromone compounds was the same as the one used for SlituOR5, SlituOR75, SlitOR5, and SlitOR75 characterization [11]. Pheromone standards were obtained from Sigma-Aldrich (St. Louis, MO, USA) and Pherobank (Wijk bij Duurstede, The Netherlands). All compounds tested were diluted in hexane. For the screening assays, 10 µl of each pheromone solution (1 µg/µl) was applied on a 1 cm^2^ piece of filter paper, which was then inserted into a Pasteur pipette to serve as the stimulus cartridge. Dose–response experiments utilized concentrations ranging from 1 ng to 100 µg loaded onto the stimulus cartridge. Neuronal responses were recorded and analyzed with pCLAMP 10 software. Net activity of at1 neurons was calculated by subtracting the spontaneous firing rate from the firing rate recorded during odorant exposure, following the procedure described in de Fouchier et al. [54]. To verify the absence of endogenous DmelOR67d expression in at1 neurons, 10 µg of cis-vaccenyl acetate (cVA)—a known ligand of DmelOR67d [26]—was applied.

Each compound and dose were tested with eight biological replicates (transgenic flies). Odorants were classified as ligands if the neuronal activation they induced was statistically significant compared to solvent controls, determined using a Friedman test followed by Dunn’s post hoc test with Benjamini and Yekutieli correction (*p* < 0.05). For dose–response experiments, data were fitted with sigmoidal curves when a plateau was reached, or with exponential functions otherwise, using the curve_fit function from the SciPy 1.16.0 library. Statistical analyses were performed using R and Python 3.11.5.

### Determination of the female calling behavior period in *S. picta*

To identify the best period for sex pheromone extraction in *S. picta* females, we observed the female calling behavior. Following eclosion, individual virgin females were housed separately in 500-mL plastic containers. Calling behavior, indicated by pheromone gland extrusion, was monitored during the first scotophase post-eclosion and in the four subsequent scotophases, corresponding to the time of the first female mortality. Observations commenced at the onset of darkness and were recorded every 30 min using a dim red light to minimize disturbance. A total of 48 females were monitored throughout the study.

### Preparation of female sex pheromone gland extracts

Sex pheromone glands of *S. picta* were dissected and extracted during the identified calling period (6 to 9 h after the onset of the scotophase, Additional file 1: Fig. S2). Virgin females, 2 days post-eclosion, were used for the procedure. To expose the pheromone gland, gentle abdominal pressure was applied to induce gland eversion. The extruded glands were carefully excised using fine microdissection scissors, briefly blotted on a filter paper to remove residual hemolymph, and transferred into 2-mL glass vials containing 1 mL of high-purity hexane. Seven biological replicates were prepared, each consisting of a single vial containing pooled glands from 20 females.

The extraction was carried out under static conditions at room temperature for 1 h. The crude extracts were purified by passing them through a glass Pasteur pipette packed with anhydrous sodium sulfate and glass wool. The eluates were subsequently concentrated to a final volume of 100 µL under a gentle stream of nitrogen. The same extracts were used for both GC-EAD and GC-MS analysis.

### GC-EAD analysis of excised male *S. picta* antennae responses to female sex pheromone extracts

The pheromone extracts above were subjected to precision concentration under controlled nitrogen stream evaporation until reaching a concentration of 4 female equivalents (FE) per microliter (4 FE/μL) for GC-EAD. The pheromone extracts were injected in a gas chromatograph (Agilent 7890B) coupled with a flame ionization detector (FID) and equipped with a DB-23 capillary column. The analysis employed a high-purity nitrogen carrier gas at a constant flow rate of 2 mL/min. The oven temperature protocol consisted of an initial 1 min hold at 40 °C, followed by a programmed temperature increase at 10 °C/min to 160 °C, then a secondary ramp at 3 °C/min to 200 °C, and a final escalation phase at 10 °C/min culminating at 250 °C. The effluent stream was split post-column in a 1:1 ratio using a heated transfer line system. Half of the effluent was delivered to a humidified air stream (400 mL/min) for antennal stimulation, while another half was directed to the FID detector. Two-day-old *S. picta* male antennae were surgically excised with micro-scissors (*n* = 18). Each antenna was mounted between two borosilicate glass microelectrodes containing a Beadle Ringer’s solution. The reference electrode was positioned at the antennal base, while the recording electrode contacted the distal segment. Electrical continuity was maintained through platinum wire connections to a Syntech EAG Combi probe, with real-time signal monitoring via the integrated software platform.

### Identification of the main female sex pheromone components of *S. picta* by GC-MS

The concentration of pheromone extracts was adjusted to 2 FE/μL for GC-MS analysis. The extracts were analyzed with an Agilent 8890 coupled to a 5977B mass selective detector equipped with a polar DB-23 column. The GC program used was the same as the one used for GC-EAD. The MS scan range was m/z 50 to 500. The pheromone components were determined by comparison of the mass spectra with the NIST 20 database, followed by further confirmation using standard compound mass spectra. Standards were purchased from Jiangsu Ninglu Biological Technology Co., Ltd. (Jiangsu Province, China) with the following purities: (*Z*,*E*)−9,11–14:OAc, 95%; (*Z*)−11–14:OAc, ≥ 90%; (*Z*)−9–14:OAc, 95%; and (*E*)−11–14:OAc, 92%. For ratio calculation, we used 11:OAc as an internal standard. Standard samples containing equal masses of each pheromone component and 11:OAc were prepared and analyzed by GC. A correction factor (*f*) for each component was calculated from the peak area ratio relative to the internal standard (Additional file 1: Table S2). Subsequently, for each insect sample, we added 500 ng of 11:OAc prior to GC analysis. Absolute amount of each pheromone component per insect was calculated using the measured peak areas and the corresponding correction factors, and the final blend ratio was determined. The calculation formula was as follows: (pheromone area)/(11:OAc area)*f*500/5 (each repeat contained 5 pheromone glands).

### EAG responses of adult male *S. picta* to synthetic compounds

EAG responses were recorded as for GC-EAD on isolated antennae of 2-day-old male *S. picta* moths. Synthetic compounds corresponding to the compounds identified in *S. picta* pheromone gland extract, along with related analogs, were prepared at two concentrations (10 ng/µL and 100 ng/µL) in hexane. A filter paper strip (2.5 × 0.75 cm) was loaded with 10 µL of each solution and inserted into a clean Pasteur pipette to serve as an odorant delivery source. Antennal preparations, instrumentation (Syntech, Germany), and recording procedures followed the established protocol outlined in Han et al. [55]. Responses were recorded from 10 individual antennae per compound and dose. A compound was considered to elicit a response if the antennal activity it induced was significantly greater than that evoked by the hexane control, as determined by a Friedman test followed by Dunn’s post hoc test with Benjamini and Yekutieli correction (*p* < 0.05). All statistical analyses were conducted using R.

### Short-range behavioral responses of *S. picta* adult males to synthetic pheromone components

After 1 h of acclimatization in the behavioral room, unmated 2-day-old males were individually transferred into plastic cups (15 cm height × 8 cm diameter). Behavioral assays were conducted 6–8 h after the onset of the scotophase under controlled conditions (25 ± 2 °C, 60 ± 10% relative humidity). A piece of filter paper fixed to the top of each cup with a needle served as the odor stimulus source.

To assess the behavioral activity of (*Z*,*E*)−9,11–14:OAc, moths were sequentially exposed to three odorant stimuli. First, a negative control (10 µg hexane) was applied to the filter paper for 5 min. After a 5-min interval with no stimulus, a new filter paper containing 10 µg of (*Z*,*E*)−9,11–14:OAc was introduced for 5 min. Following another 5-min pause, a positive control was presented for 5 min that consisted of a piece of filter paper freshly rubbed with an excised sex pheromone gland of a 2-day-old virgin female. During each 5-min stimulus period, three stereotypical male sexual behaviors previously reported in the genus *Spodoptera* were recorded: wing-fanning, hair-pencil display, and odor-source contact [29–31]. Males that failed to respond to the positive control were excluded from the analysis. In total, responses from 16 males were evaluated. A parallel experiment was conducted by replacing (*Z*,*E*)−9,11–14:OAc with a synthetic blend of (*Z*,*E*)−9,11–14:OAc, (*Z*)−9–14:OAc, (*E*)−11–14:OAc, and (*Z*)−11–14:OAc that contained a total of 10 µg of compounds in the relative ratio 100:45:34:3. In this experiment, the behavioral responses of 17 males were recorded. (*Z*,*E*)−9,11–14:OAc, the synthetic blend, and the positive control were considered active if the proportion of males exhibiting any of the three recorded behaviors (wing‑fanning, hair‑pencil display, or odor-source contact) was significantly higher than that observed in response to the hexane control. Statistical significance was determined using a generalized linear mixed model (response ~ TESTED COMPOUND × STEREOTYPICAL SEXUAL BEHAVIORS + (1|Male_ID)), followed by multiple-comparison post hoc tests with Bonferroni correction. All statistical analyses were conducted using R.

## Supplementary Information

Additional file 1: Fig. S1. Sequence alignment and binding region properties. Fig. S2. Calling behavior and GC-EAD. Fig. S3. EAG. Fig. S4. Sexual behavior. Fig. S5. AlphaFold 3 models. Table S1. Primer sequences for RT-qPCR. Table S2. Pheromone component ratio calculation.

## Data Availability

The experimental data from single-sensillum recordings were deposited on GitHub (https://github.com/chemosim-lab/Data_Phero_SpicOR5-75). Raw data are accessible in this repository, and all other data are available from the corresponding authors upon reasonable request.

## Abbreviations

cVA: Cis-vaccenyl acetate
Dmel: *Drosophila melanogaster*
EAG: Electroantennography
EF1-α: Elongation factor 1-alpha
FE: Female equivalent
FID: Flame ionization detector
GC-MS: Gas chromatography–mass spectrometry
GC-EAD: Gas chromatography–electroantennographic detection
OR: Odorant receptor
OSN: Olfactory sensory neuron
PLDDT: Predicted local distance difference test
RMSD: Root-mean-square deviation
RT-qPCR: Quantitative real-time PCR
Sexi: *Spodoptera exigua*
Sfru: *Spodoptera frugiperda*
Slit: *Spodoptera littoralis*
Slitu: *Spodoptera litura*
Spic: *Spodoptera picta*
(*Z*,*E*)-9,11–14:OAc: (*Z*,*E*)-9,11-Tetradecadienyl acetate
