# Supplementary material for: Odorant receptor structures predict the major female sex pheromone component in a moth
Source: BMC Biol. 2026 Sep 21;24:193. doi: 10.1186/s12915-026-02717-1 (PMC13591803; doi:10.1186/s12915-026-02717-1)
Supplement: Supplementary file 1 [file 12915_2026_2717_MOESM1_ESM.docx]

**Additional file**

**Odorant receptor structures predict the major female sex pheromone component in a moth**

Arthur Comte^1,2^, Zhiqiang Wei^3^, Hui Li^3^, Wen-Zhe Xing^3^, Riccardo Moracci^2^, Jin Zhang^3^*, Sébastien Fiorucci^1^*, Emmanuelle Jacquin-Joly^2^*

* corresponding authors: jinzhang001@njau.edu.cn, sebastien.fiorucci@univ-cotedazur.fr, emmanuelle.joly@inrae.fr

^1^ Université Côte d’Azur, CNRS, ICN, France

^2^ INRAE, Sorbonne Université, CNRS, IRD, Université Paris Cité, Université Paris Est Créteil Val de Marne, Institut d’Ecologie et des Sciences de l’Environnement de Paris (iEES-Paris), Versailles, France

^3^ Department of Entomology, College of Plant Protection, Nanjing Agricultural University, Nanjing, China

**Figure S1.** **OR5 orthologs and paralogs across the five studied *Spodoptera* species form two distinct clusters based on binding region physicochemical properties. (a)** Multiple sequence alignment of SpicOR5 and SpicOR75 with orthologous sequences from *S. litura, S. littoralis, S. frugiperda*, and *S. exigua*, generated using the MAFFT web server. Predicted transmembrane domains (TM) are indicated by black boxes. Fully conserved residues across all eight sequences are denoted by asterisks, while partially conserved residues are indicated by dots. Amino acids predicted to contribute to the binding region are colored red. **(b)** Clustering of OR5 orthologs and paralogs based on predicted binding region volume and the percentage of apolar spheres within it, calculated using Fpocket. Clustering was performed with HDBSCAN; clustered PRs are shown in color, while unclustered proteins are shown in grey.

**Figure S2. *S. picta* adult females exhibit peak calling behavior during the first two days after adult emergence, occurring between 6 and 9 hours after the onset of the scotophase. One component present in the female sex pheromone extract of *S. picta* elicited electrophysiological responses in adult male antennae. (a)** Percentage of females exhibiting calling behavior during the first five scotophases post-emergence (n = 48), recorded at 30-minute intervals. Curves are shaded from light grey to black to represent increasing female age. The red curve corresponds to the age selected for pheromone extraction. **(b)** GC-EAG recordings. Three representative responses of excised *S. picta* adult male antennae are presented (one color per antennae, top) in response to the GC effluent of a typical female sex pheromone extract (down). The only component that elicited a consistent response is indicated by an arrow and has been further identified as (*Z,E*)-9,11-14:OAc.

**Figure S3. Among the compounds tested, (*Z,E*)-9,11-14:OAc elicited the strongest antennal response in male *S. picta*.** Box plots show EAG responses of male *S. picta* antennae to 1000 ng of the compounds identified in the female sex pheromone gland extracts (colored as in Figure 4a) and structurally related analogs (in black) (*n* = 10). Whiskers extend to 1.5 times the interquartile range from the quartiles, with outliers indicated by dots. Asterisks denote statistically significant differences between responses to the odorant and the solvent (Friedman test followed by Dunn’s post hoc test with Benjamini & Yekutieli correction for multiple comparisons; **adjusted *p* < 0.01, *** adjusted *p* < 0.001).

**Figure S4.** **Synthetic blend elicits strong adult male sexual behavior.** Bar chart summarizing the male behavior to a synthetic blend (10 µg in total on filter paper) of (*Z,E*)-9,11-14:OAc, (*Z*)-9-14:OAc, (*E*)-11-14:OAc, and (*Z*)-11-14:OAc in the relative ratio 100:45:34:3. Three behaviors were tracked to measure the attraction of males: wing-fanning (white bars), hair-pencil displaying (light grey bars), and filter paper touching (dark grey bars). Values represent the percentage of responding males (n = 17). Hexane was used as a negative control (10 µg on filter paper) and a female gland extract as a positive control (one gland excised and applied to filter paper). Asterisks illustrate statistically significant differences between the synthetic blend or female gland extract and the negative control (generalized linear mixed model: response ~ compound tested  × stereotypical sexual behaviors + (1|Male_ID), followed by multiple-comparison post hoc tests with a Bonferroni correction; * adjusted *p* < 0.05, ** adjusted *p* < 0.01, *** adjusted *p* < 0.001).

**Figure S5. AlphaFold 3 models of OR5 orthologs and paralogs across the five examined *Spodoptera* species exhibit high structural confidence, particularly within the predicted binding regions. (a)** pLDDT scores for top-ranked AlphaFold 3 models of OR5 orthologs and paralogs. Scores were calculated for both the full-length protein sequences and the predicted binding regions. **(b)** Top-ranked AlphaFold 3 models of SpicOR5 and SpicOR75, colored by per-residue pLDDT scores: blue (≥90), light blue (85–89), yellow (70–84), and red (<70), corresponding to very high, high, confident, and low prediction accuracy, respectively. Side chains of residues within the predicted binding regions are shown as sticks. 3D-models were visualized using PyMOL.

**Table S1. Primer sequences for RT-qPCR.**

| **Primer name** | **Primer sequence (5’->3’)** |
| --- | --- |
| SpicOR5-Forward | ATACACAGCATCATCTACCA |
| SpicOR5-Reverse | GGATCAGTAAGACGCAGTA |
| SpicOR75-Forward | CGAGCACTCAGACCTATT |
| SpicOR75-Reverse | CCACATGGCATTCATCAG |
| EF1α-Forward | ATCGGTGGTATTGGTACG |
| EF1α-Reverse | GACTTCAGTGGTGATGTTG |

Sequence of *S. picta* elongation factor EF1α

ATGGGCAAGGAAAAGATTCACATTAACATTGTCGTCATTGGACACGTCGACTCCGGCAAGTCCACCACCACCGGTCACTTGATCTACAAATGCGGTGGTATCGACAAACGTACCATCGAGAAGTTCGAGAAGGAGGCCCAGGAAATGGGTAAGGGTTCCTTCAAATATGCCTGGGTATTGGACAAACTGAAGGCTGAGCGTGAACGTGGTATCACCATTGATATTGCTCTGTGGAAGTTCGAAACCGCTAAATACTATGTCACCATTATTGACGCTCCCGGACACAGAGATTTCATCAAGAACATGATCACTGGAACCTCCCAGGCTGATTGCGCTGTACTCATTGTCGCCGCTGGTACCGGTGAATTCGAGGCTGGTATCTCGAAGAACGGACAGACCCGTGAGCACGCTCTGCTCGCTTTCACACTCGGTGTCAAGCAGCTGATTGTGGGCGTCAACAAAATGGACTCCACTGAGCCCCCATACAGCGAATCCCGTTTCGAGGAAATCAAGAAGGAAGTGTCCTCCTACATCAAGAAGATCGGTTACAACCCAGCTGCTGTCGCTTTCGTACCCATTTCTGGCTGGCACGGAGACAACATGTTGGAGGCCTCAACCAAAATGCCCTGGTTTAAGGGATGGAACGTTGAGCGCAAGGAAGGCAAGGCTGAAGGTAAATGCCTCATTGAGGCCCTCGACGCCATCCTGCCCCCTGCTCGCCCCACAGACAAGCCCCTGCGTCTTCCCCTCCAGGACGTATACAAAATCGGTGGTATTGGTACGGTGCCCGTAGGCAGAGTTGAAACTGGTATCCTCAAGCCTGGTACCATCGTCGTCTTCGCCCCTGCCAACATCACCACTGAAGTCAAGTCTGTGGAGATGCACCACGAAGCTCTCCAAGAGGCCGTACCCGGTGACAACGTTGGTTTCAACGTAAAGAACGTTTCCGTCAAGGAGTTGCGTCGTGGTTACGTCGCTGGTGACTCCAAGAACAACCCACCCAAGGGCGCCGCCGATTTCACAGCACAGGTCATCGTGCTCAACCACCCTGGTCAAATCTCAAACGGATACACACCTGTGCTGGATTGCCACACAGCCCACATTGCCTGCAAGTTCGCTGAAATCAAAGAGAAGGTTGACCGTCGTACTGGTAAATCCACTGAAGACAACCCTAAATCCATCAAATCTGGAGATGCCGCCATCGTCAACTTGGTTCCCTCCAAGCCTCTGTGTGTTGAGTCCTTCCAGGAATTCCCTCCCCTCGGTCGTTTCGCCGTGCGTGACATGAGGCAGACGGTCGCTGTGGGTGTAATCAAGGCTGTCAACTTCAAGGAAGCTGGTGGTGGTAAGGTCACCAAAGCCGCCGAGAAGGCCACCAAGGGCAAGAAGTAG

**Table S2. Pheromone component ratio calculation.** 11:OAc was used as internal standard. Correction factors (f) were calculated from peak area ratios after GC analysis of standard mixtures containing equal masses of each component and 11:OAc, with 11:OAc set as f = 1. For insect samples, 500 ng of 11:OAc was added prior to GC analysis. Absolute amounts per insect were calculated as: (pheromone area)/(11Ac area)*f*500/5 ng, where 5 corresponds to the number of sex pheromone glands pooled per replicate.

|  | **11:OAc** | **(Z)-9-14:OAc** | **(E)-11-14:OAc** | **(Z)-11-14:OAc** | **(Z,E)-9,11-14:OAc** |
| --- | --- | --- | --- | --- | --- |
| **Peak Area** | 49469642 | 48984375.28 | 51696846 | 43840985.22 | 47469543.1 |
| **Correction factor (f)** | 1 | 1.009906561 | 0.956917991 | 1.128388008 | 1.042134361 |

| **GC** | **Name** | **RT** | **Area** |
| --- | --- | --- | --- |
| Repeat 1 | 11:OAc | 14.572 | 7499004.93 |
|  | (Z)-9-14:OAc | 20.306 | 146922.08 |
|  | (E)-11-14:OAc | 20.464 | 2142202.93 |
|  | (Z)-11-14:OAc | 20.587 | 1355531.68 |
|  | (Z,E)-9,11-14:OAc | 21.449 | 3822039.61 |
| Repeat 2 | 11:OAc | 14.578 | 13657064.04 |
|  | (Z)-9-14:OAc | 20.306 | 294879.01 |
|  | (E)-11-14:OAc | 20.47 | 3942611.8 |
|  | (Z)-11-14:OAc | 20.587 | 2816177.78 |
|  | (Z,E)-9,11-14:OAc | 21.456 | 8970381.05 |
| Repeat 3 | 11:OAc | 14.572 | 7132404.89 |
|  | (Z)-9-14:OAc | 20.313 | 101355.43 |
|  | (E)-11-14:OAc | 20.471 | 1534993.96 |
|  | (Z)-11-14:OAc | 20.594 | 785946.21 |
|  | (Z,E)-9,11-14:OAc | 21.449 | 3110386.31 |

| **Calculate** | **f** | **Repeat 1** | **Repeat 2** | **Repeat 3** | **Mean（ng/FE)** | **Rate** |
| --- | --- | --- | --- | --- | --- | --- |
| 11:OAc (500 ng) |  |  |  |  |  |  |
| Z9-14:OAc | 1.00990656 | 1.978630151 | 2.180558326 | 1.435133245 | 1.864773907 | 0.033 |
| (E)-11-14:OAc | 0.95691799 | 27.33579378 | 27.62494303 | 20.59422254 | 25.18498645 | 0.452 |
| (Z)-11-14:OAc | 1.12838801 | 20.39691541 | 23.26811404 | 12.43412695 | 18.6997188 | 0.336 |
| (Z,E)-9,11-14:OAc | 1.04213436 | 53.1147645 | 68.45060036 | 45.44666913 | 55.670678 | 1.000 |

***** Calculation formula: (pheromone area)/(11Ac area)*f*500/5

5: each repeat contains 5 sex pheromone glands
